# Supplementary material for: Single-cell transcriptomics analysis of bullous pemphigoid unveils immune-stromal crosstalk in type 2 inflammatory disease
Source: Nat Commun. 2024 Jul 15;15:5949. doi: 10.1038/s41467-024-50283-3 (PMC11251189; doi:10.1038/s41467-024-50283-3)
Supplement: Supplementary file 1 — Supplementary Information [file 41467_2024_50283_MOESM1_ESM.pdf]

Single-cell transcriptomics analysis of bullous pemphigoid unveils immune-stromal crosstalk in type 2 inflammatory disease

Tingting Liu[1,2,6], Zhenzhen Wang[1,2,3,6], Xiaotong Xue[1,2], Zhe Wang[1,2], Yuan Zhang[1,2], Zihao Mi[1,2], Qing Zhao[1,2], Lele Sun[1,2], Chuan Wang[1,2], Peidian Shi[1,2], Gongqi Yu[1,2], Meng Wang[1,2], Yonghu Sun[1,2], Fuzhong Xue[3], Hong Liu[1,2,5*], Furen Zhang[1,2,4,5*]

[1]Hospital for Skin Diseases, Shandong First Medical University, Jinan, Shandong, China

[2]Shandong Provincial Institute of Dermatology and Venereology, Shandong Academy of Medical Sciences, Jinan, Shandong, China

[3]Department of Biostatistics, School of Public Health, Cheeloo College of Medicine, Shandong University, Jinan, Shandong, China;

[4]Shandong University of Traditional Chinese Medicine, Jinan, Shandong, China;

[5]School of Public Health, Shandong First Medical University and Shandong Academy of Medical Sciences, Jinan, Shandong, China.

[6]These authors contributed equally: Tingting Liu and Zhenzhen Wang.

*Corresponding author, these authors jointly supervised this work: Furen Zhang and Hong Liu.

Furen Zhang, MD, PhD

27397, Jingshi Rd, Jinan City, Shandong, China.

Phone: +86-053187298801

E-mail: zhangfuren@hotmail.com;

Hong Liu, MD, PhD

27397, Jingshi Rd, Jinan City, Shandong, China.

Phone: +86-053187298870

E-mail: hongyue2519@hotmail.com

Supplementary information

Supplementary Fig. 1 The quality and clustering of the skin scRNA-seq data.

Supplementary Fig. 2 Sub-clustering of the skin immune cluster.

Supplementary Fig. 3 Sub-clustering of the fibroblasts and keratinocytes clusters.

Supplementary Fig. 4 The expression and role of PLA2G2A.

Supplementary Fig. 5 All the significant ligand-receptor pairs that contribute to the signaling sending from fibroblast sub-populations to other subsets by CellChat analysis.

Supplementary Fig. 6 The expression profile of *CXCL12* and *CXCR4* in skin cells.

Supplementary Fig. 7 The analysis of significant mean top50 interacting pair from Th2 and Proliferating T cells by CellPhoneDB.

Supplementary Fig. 8 The expression profile of *CCL17* and *CCR4* in immune cells.

Supplementary Fig. 9 PLA2G2A and CCL17 elevate the frequency of plasma cells.

Supplementary Fig. 10 The quality of PBMC and blister samples, as well as the identification of clusters within PBMC samples.

Supplementary Fig. 11 The Dot plot of the signature genes of PBMC clusters and the distribution of clusters within the PBMC samples.

Supplementary Fig. 12 The distribution of clusters in blister samples and the *IL13-IL13RA1* ligand-receptor pair in PBMCs and blister.

Supplementary Fig. 13 *CCL17-CCR4* ligand-receptor pair in PBMC and blister.

Supplementary Fig. 14 *CCL19-CCR7* ligand-receptor pair in skin and blister samples.

Supplementary Fig. 15 The gating strategy of T cells and B cells to test the expression of CXCR4, CCR4 and CD138.

Supplementary Table 1 The sample information included in the study.

Supplementary Table 2 The detailed information of antibodies used in the study.

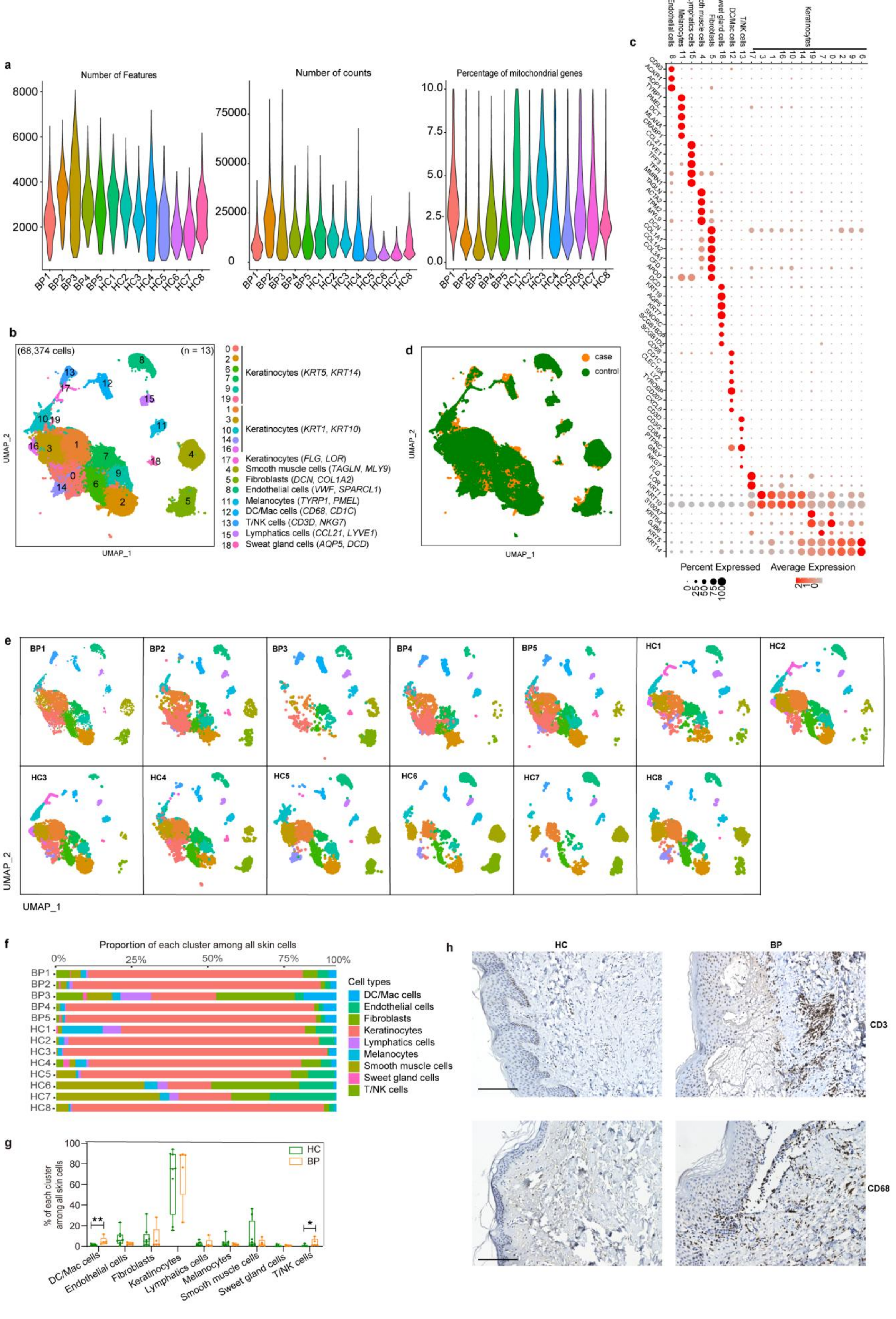
a
Number of Features
Number of counts
Percentage of mitochondrial genes
BP1 BP2 BP3 BP4 BP5 HC1 HC2 HC3 HC4 HC5 HC6 HC7 HC8
b
(68,374 cells)
(n = 13)
Keratinocytes (KRT5, KRT14)
Keratinocytes (KRT1, KRT10)
Keratinocytes (FLG, LOR)
Smooth muscle cells (TAGLN, MLY9)
Fibroblasts (DCN, COL1A2)
Endothelial cells (VWF, SPARCL1)
Melanocytes (TYRP1, PMEL)
DC/Mac cells (CD68, CD1C)
T/NK cells (CD3D, NKG7)
Lymphatics cells (CCL21, LYVE1)
Sweat gland cells (AQP5, DCD)
UMAP_1
UMAP_2
c
Endothelial cells
Melanocytes
Lymphatics cells
Smooth muscle cells
Fibroblasts
Sweet gland cells
DC/Mac cells
T/NK cells
Keratinocytes
Percent Expressed
Average Expression
d
case
control
e
f
Proportion of each cluster among all skin cells
Cell types
DC/Mac cells
Endothelial cells
Fibroblasts
Keratinocytes
Lymphatics cells
Melanocytes
Smooth muscle cells
Sweet gland cells
T/NK cells
g
% of each cluster among all skin cells
HC
BP
h
HC
BP
CD3
CD68

Supplementary Fig. 1 The quality and clustering of the skin scRNA-seq data

a Violin plots illustrating the quality control metrics used in the scRNA-seq analysis for each skin sample's features, counts, and mitochondrial percentage per cell. **b** UMAP plot for 68,374 cells from skin samples of the discovery cohort, the signature genes for each cell type were indicated in parentheses. **c** Dot plot of the signature genes for the identification of major skin cell types. **d** UMAP plot for skin cells split by bullous pemphigoid (BP) and healthy controls (HC). Cells from BP patients are shown in orange, whereas cells from HC are shown in green. **e** UMAP plot for skin cells split by each sample. **f** Bar plots illustrate the relative contributions of skin cell types in each sample. **g** Comparison of the percentage of each cell type in all skin cells between BP patients and HC. Each dot represented a donor. BP patients (n = 5) and HC (n = 8). *P*-values were calculated using two-sided Mann-Whitney U-test where ** = *P*-value = 0.005 and * = *P*-value = 0.013, only *P*-values<0.05 are shown. Minima: Lower limit of the whisker. Maxima: Upper limit of the whisker. Centre: Median line inside the box. The upper and lower box bounds represent the 25% and 75% percentile of data. **h** Immunochemistry staining showing the increase of $CD3^+$ T cells and $CD68^+$ macrophages within the lesion of BP patients. Scale bar = 150 μm. Data are from at least three independent experiments in **h**.

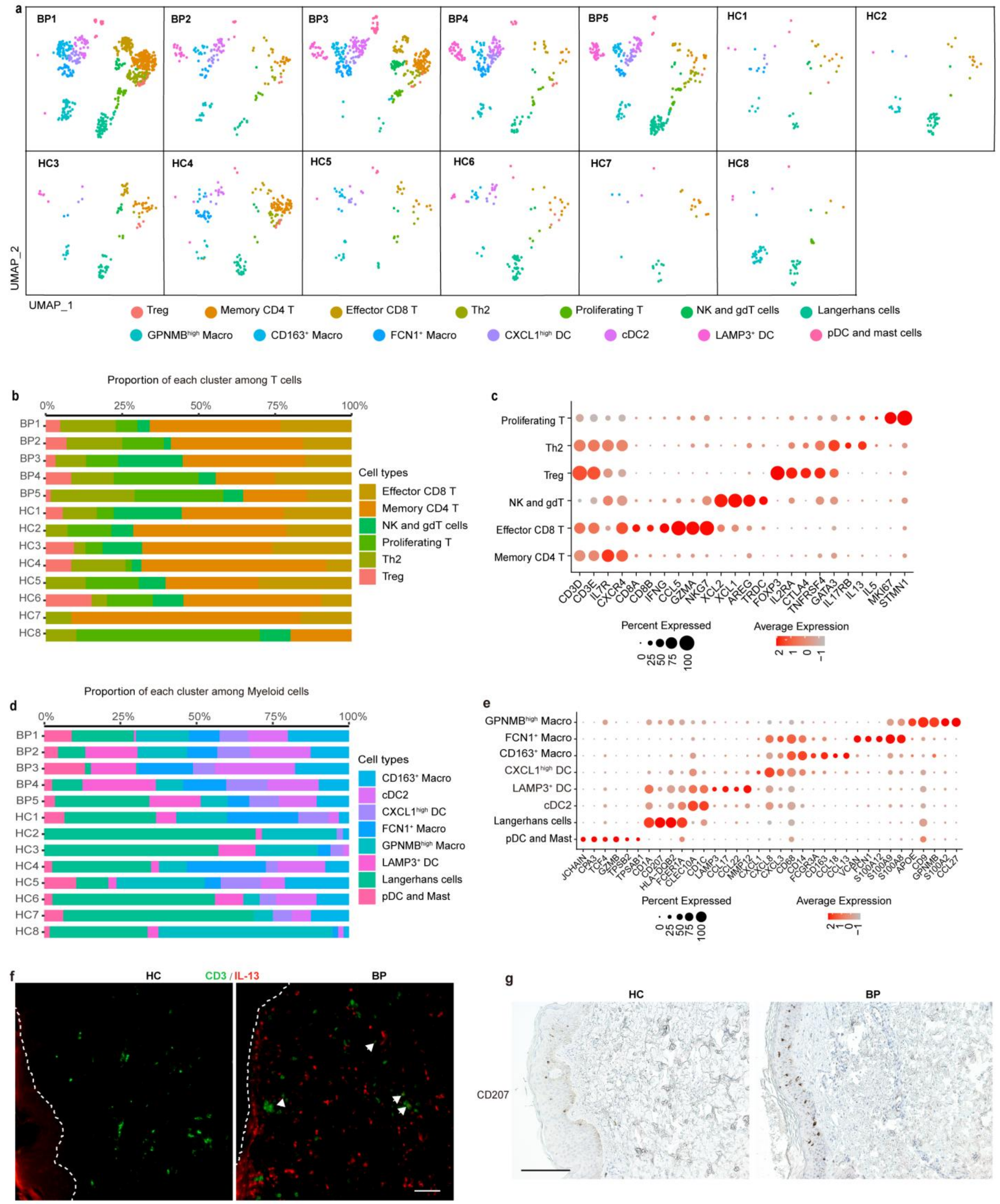

Supplementary Fig. 2 Sub-clustering of the skin immune cluster.

a UMAP plot for skin immune cells split by each sample. **b** Bar plots illustrate the relative contributions of skin T cell types in each sample. **c** Expressions of major discriminative marker

genes in skin T cells. **d** Bar plots illustrate the relative contributions of skin myeloid cell types in each sample. **e** Expressions of major discriminative marker genes in skin myeloid cells. **f** Immunofluorescence co-staining of IL-13 and CD3 further confirmed the increase of Th2 cells in BP lesions, scale bar = 50 μm. **g** The immunochemistry stainings show the infiltration of Langerhans cells, scale bar = 150 μm. Data are from at least three independent experiments in **f** and **g**.

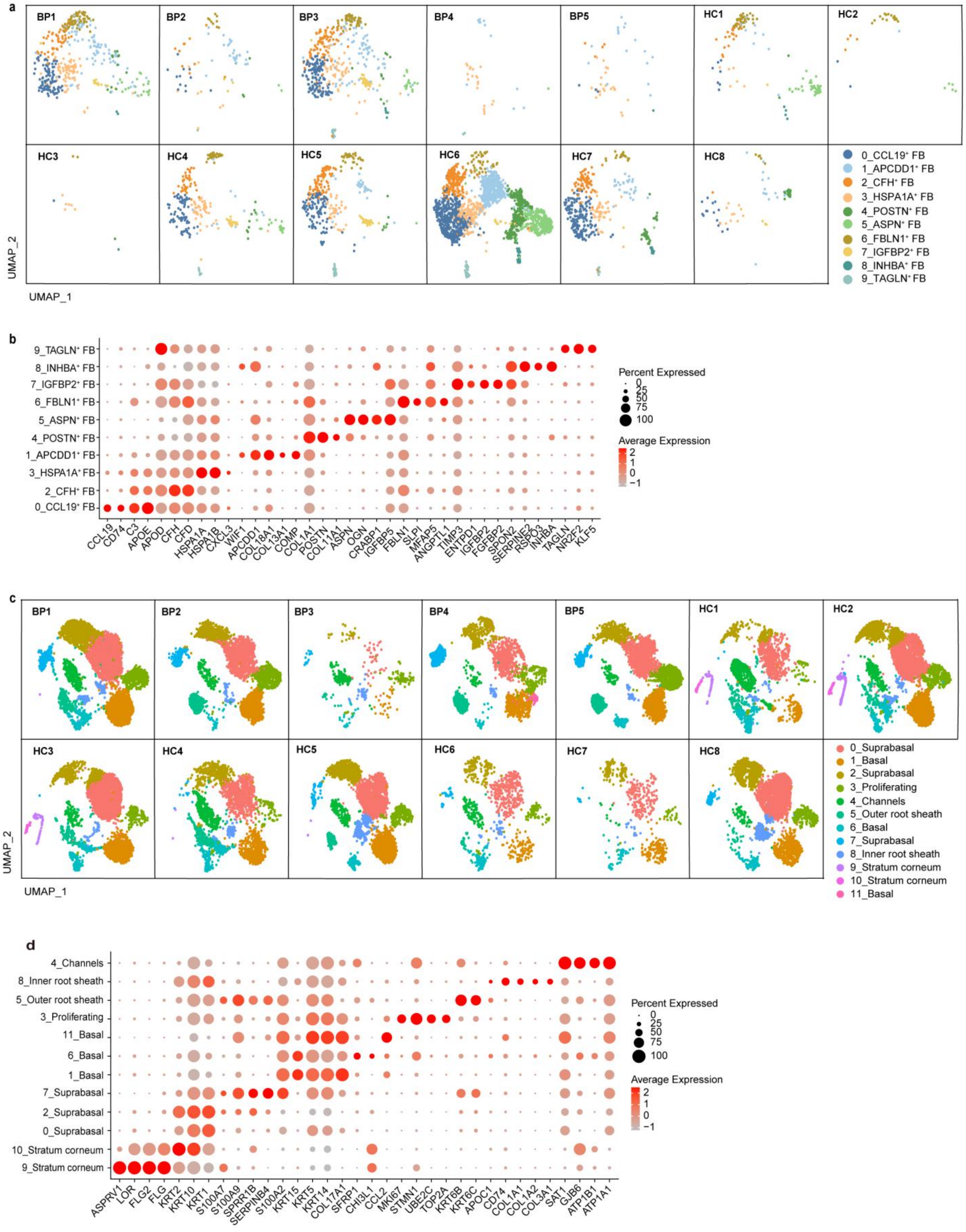

Supplementary Fig. 3 Sub-clustering of the fibroblasts and keratinocytes clusters.

a UMAP plot for fibroblasts sub-clustering split by each sample. **b** Expressions of major discriminative marker genes in fibroblasts sub-clustering. **c** UMAP plot for keratinocytes sub-clustering split by each sample. **d** Expressions of major discriminative marker genes in keratinocytes sub-clustering.

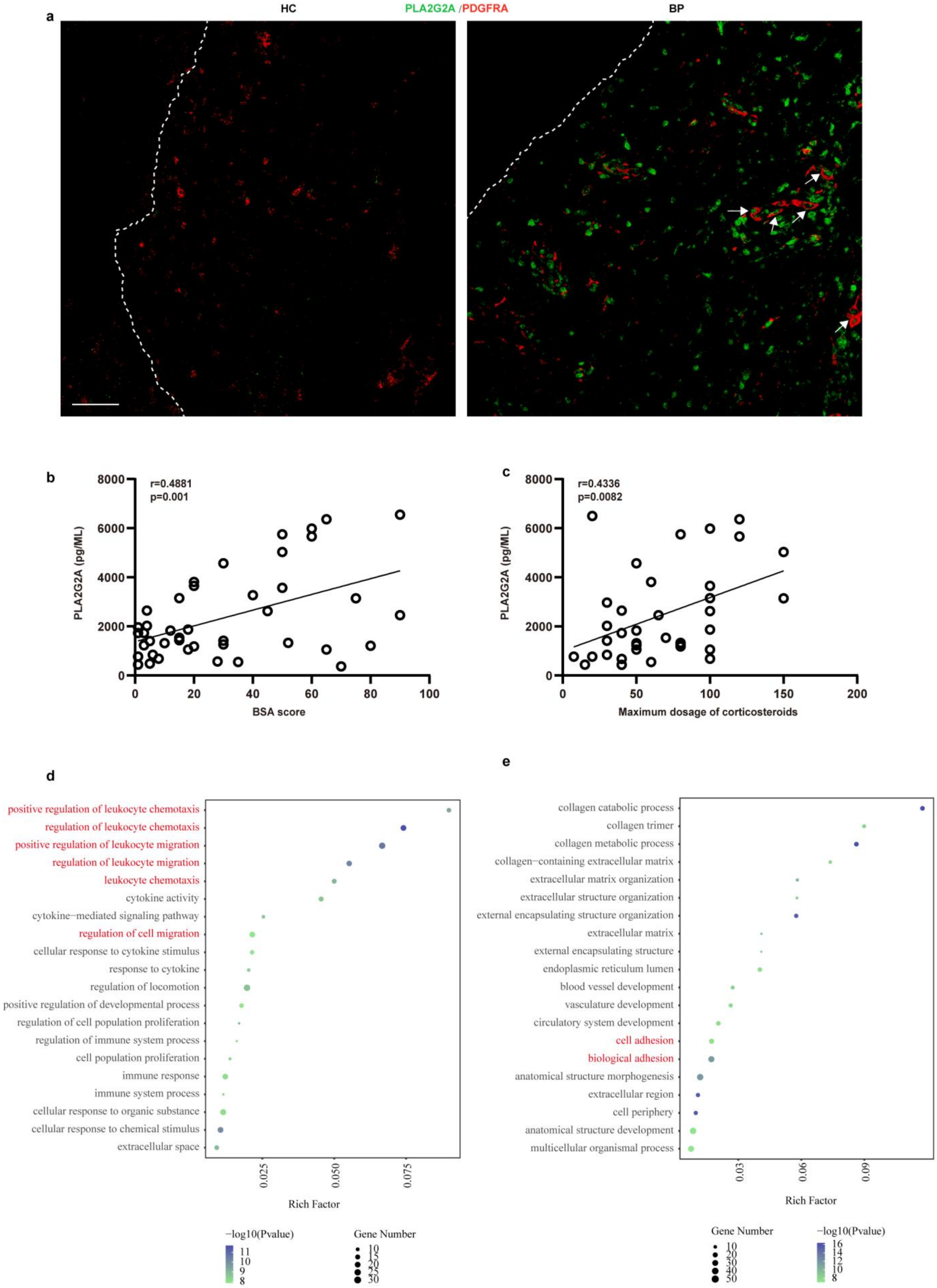

Supplementary Fig. 4 The expression and role of PLA2G2A.

a Immunofluorescence co-staining of fibroblast marker PDGFRA and PLA2G2A showing the elevation of PLA2G2A in fibroblasts from BP lesions, scale bar = 50 μm. **b** The positive correlation between the level of serum PLA2G2A and BSA score in BP patients (n = 42). **c** The positive correlation between serum PLA2G2A and the maximum dosage of corticosteroids in BP patients (n = 36). *P*-values in **b** and **c** were calculated using two-sided Pearson correlation test. r-values were Pearson correlation coefficients. **d** The enrichment of positive regulation of leukocyte chemotaxis, positive regulation of leukocyte migration and regulation of cell migration in *CCL19*$^+$ FB cells. **e** The enrichment of cell adhesion and biological adhesion in *APCDD1*$^+$ FB cells. The correlations in **b** and **c** were measured using the Pearson correlation coefficient. Data are from at least three independent experiments in **a**.

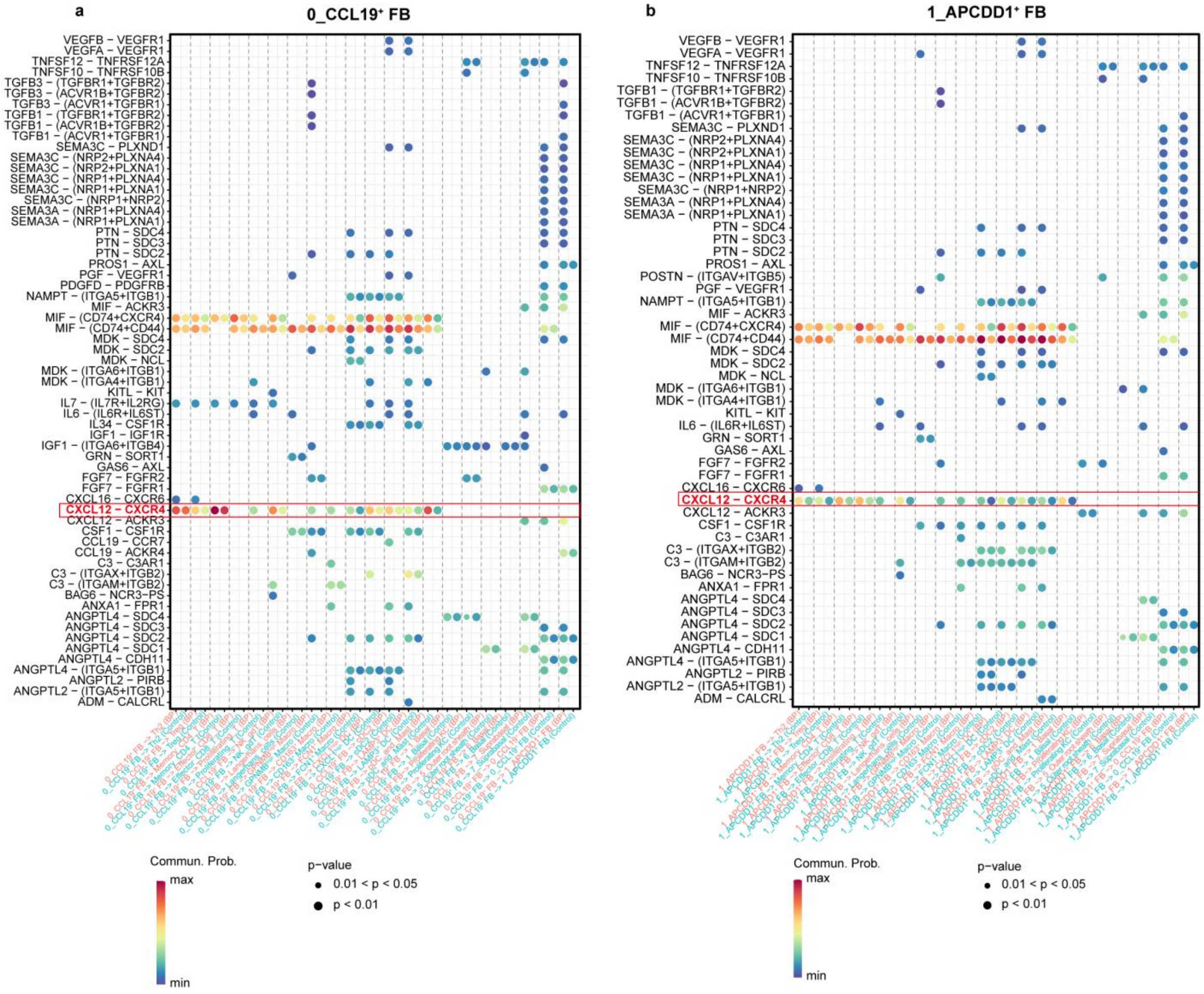

Supplementary Fig. 5 All the significant ligand-receptor pairs that contribute to the signaling sending from fibroblast subpopulations to other subsets by CellChat analysis.

a-**b** The increased ligand-receptor pairs sending from *CCL19*⁺ FB cells **(a)** and *APCDD1*⁺ FB cells **(b)**.

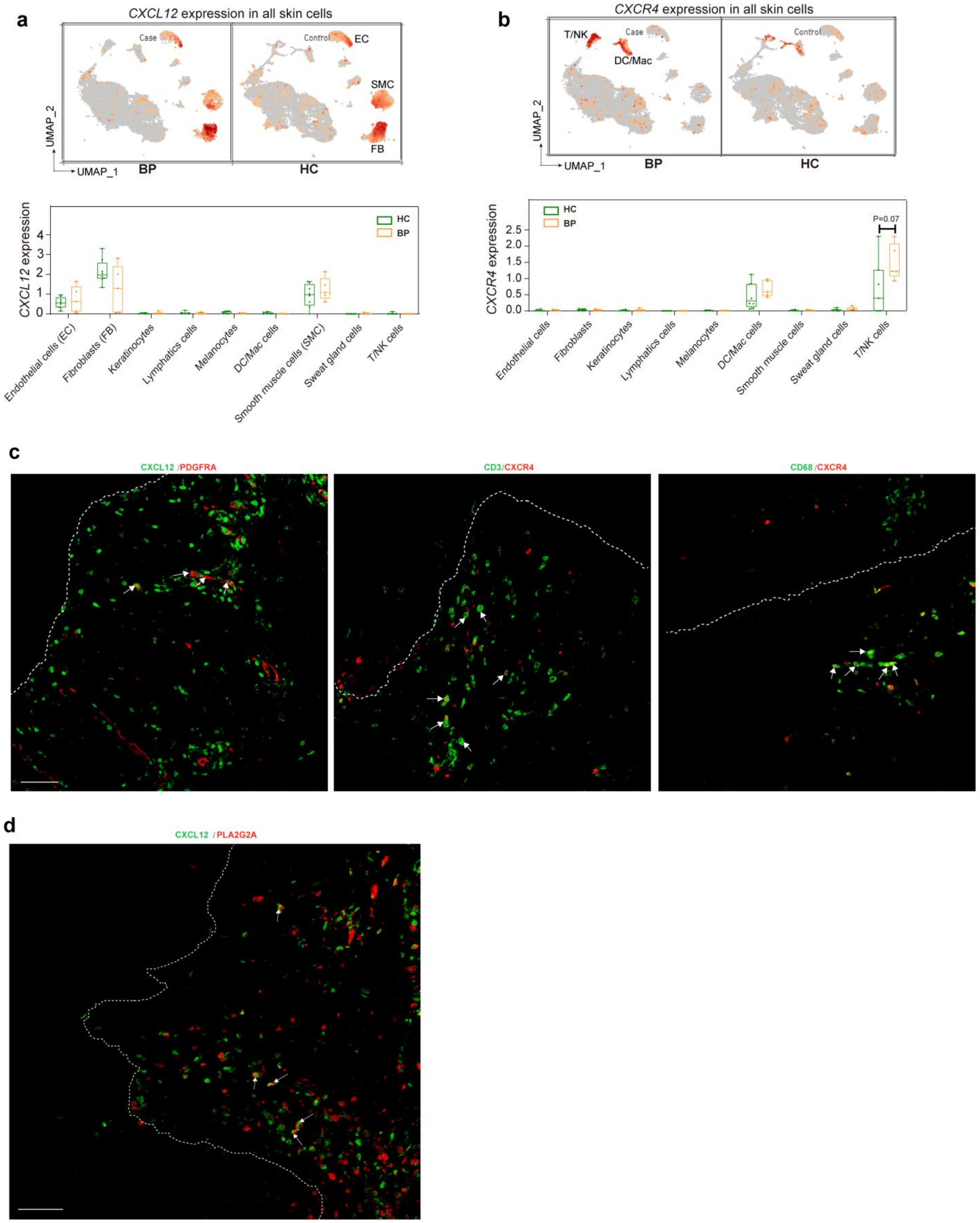

Supplementary Fig. 6 The expression profile of *CXCL12* and *CXCR4* in skin cells.

a-b The feature plot and the box plot showing *CXCL12* (**a**) and *CXCR4* (**b**) mRNA expression within skin cells. BP patients (n = 5) and HC (n = 8). *P*-values were calculated using two-sided

Mann-Whitney U-test, only *P*-values<0.05 are shown. **c** Immunofluorescence co-staining showing CXCL12 expression in *PDGFRA*$^{+}$ fibroblasts, and CXCR4 on CD3^{+} T cells and CD68^{+} macrophages, scale bar = 50 µm. **d** Immunofluorescence co-staining showing the co-expression of CXCL12 and PLA2G2A, scale bar = 50 µm. Data are from at least three independent experiments in **c** and **d**. In the box plot **a** and **b**: Minima: Lower limit of the whisker. Maxima: Upper limit of the whisker. Centre: Median line inside the box. The upper and lower box bounds represent the 25% and 75% percentile of data.

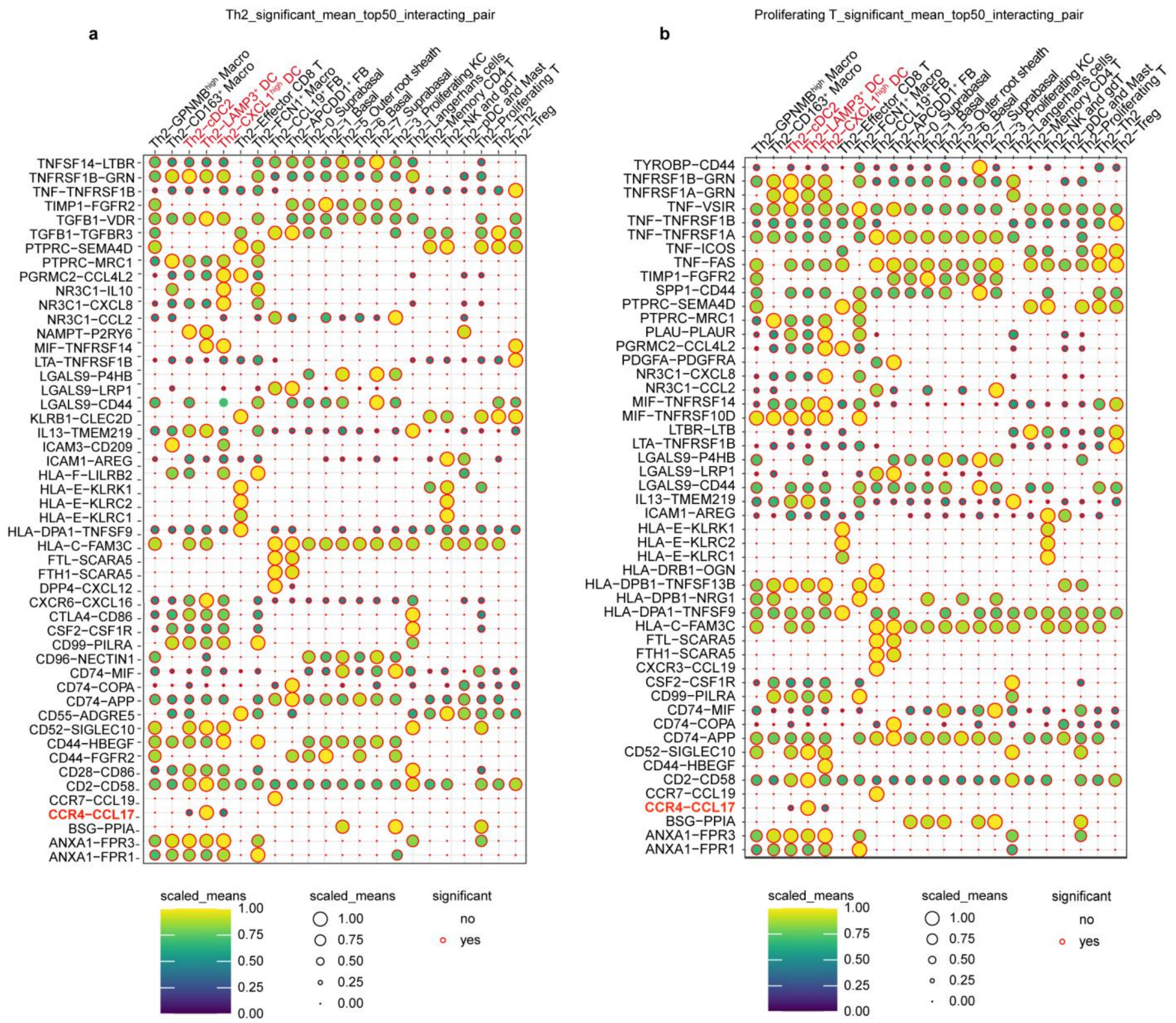

Supplementary Fig. 7 The analysis of significant mean top50 interacting pair from Th2 and Proliferating T cells by CellPhoneDB.

a-**b** The significant mean top50 interacting pairs from Th2 **(a)** and Proliferating T cells **(b)**.

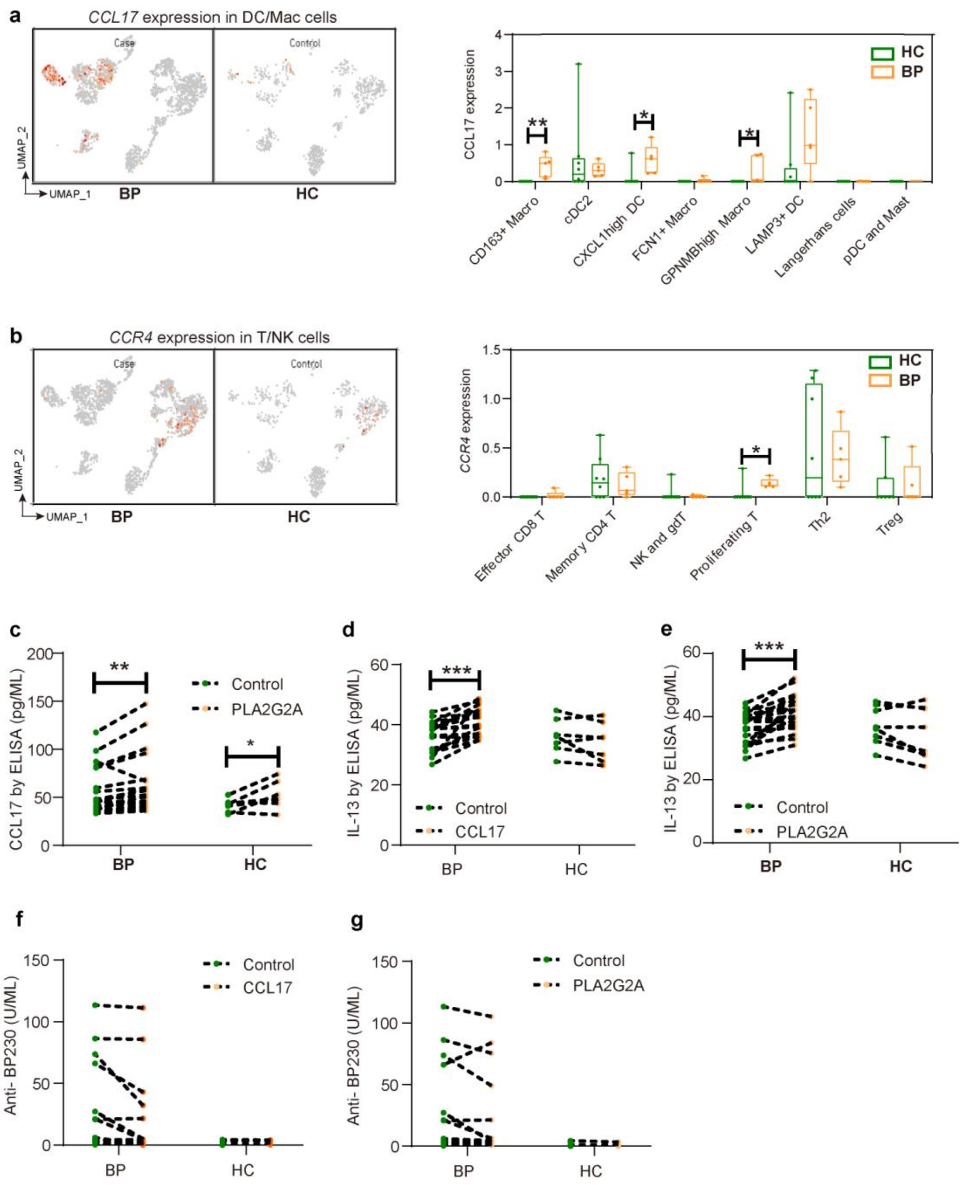

Supplementary Fig. 8 The expression profile of *CCL17* and *CCR4* in immune cells.

a The feature plot and the box plot showing *CCL17* mRNA expression within immune cells. BP patients (n = 5) and HC (n = 8). *P*-values were calculated using two-sided Mann-Whitney U-test where ** = *P*-value = 0.001, * = *P*-value = 0.011 in CXCL1^{high} DC and 0.018 in GPNMB^{high} Macro, only *P*-values<0.05 are shown. **b** The feature plot and the box plot showing *CCR4* mRNA expression within immune cells. BP patients (n = 5) and HC (n = 8). *P*-values were calculated using two-sided Mann-Whitney U-test where * = *P*-value = 0.017, only *P*-values<0.05 are shown. **c** The CCL17 secretion by sorted DCs stimulated by PLA2G2A from both BP

patients (n = 22) and controls (n = 7). **d** The IL-13 secretion by sorted T cell stimulated by CCL17 from both BP patients (n = 21) and controls (n = 7). **e** The IL-13 secretion by sorted T cell stimulated by PLA2G2A from both BP patients (n = 22) and controls (n = 8). ELISA analysis of anti-BP230 antibody titers in supernatants of CCL17 (**f**) or PLA2G2A (**g**) stimulated PBMCs from BP patients (n = 23) and HC (n = 8). *P*-values in **c-g** were calculated using paired two-sided Student's *t*-test where ** = *P*-value = 0.0012 and * = *P*-value = 0.0436 in (c), *** = *P*-value = 0.0002 in (d) and *** = *P*-value = 0.0001 in (e), only *P*-values<0.05 are shown. In the box plot **a** and **b**: Minima: Lower limit of the whisker. Maxima: Upper limit of the whisker. Centre: Median line inside the box. The upper and lower box bounds represent the 25% and 75% percentile of data.

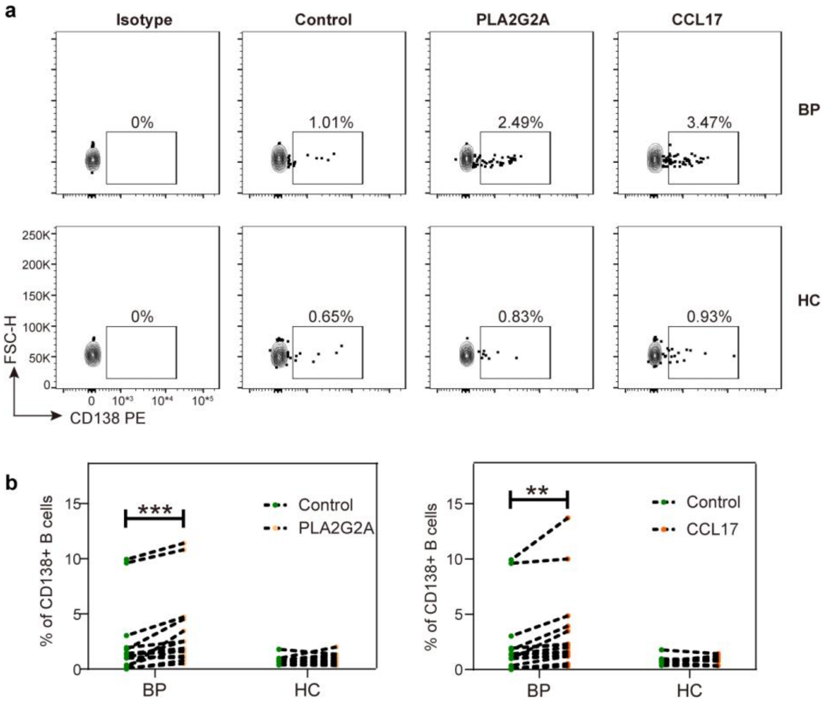

Supplementary Fig. 9 PLA2G2A and CCL17 elevate the frequency of plasma cells.

a Representative gating strategy to analyze plasma cells treated by PLA2G2A or CCL17 in the peripheral blood of BP patients and healthy controls. Plasma cells were identified as the $CD138^{+}$ cells gate on $CD19^{+}$ B cells. **b** Percentage of plasma cells in BP patients (n = 16) and HC (n = 10). *P*-values were calculated using paired two-sided Student's *t*-test where *** = *P*-value = 0.0004 in the left and ** = *P*-value = 0.0036 in the right, only *P*-values<0.05 are shown.

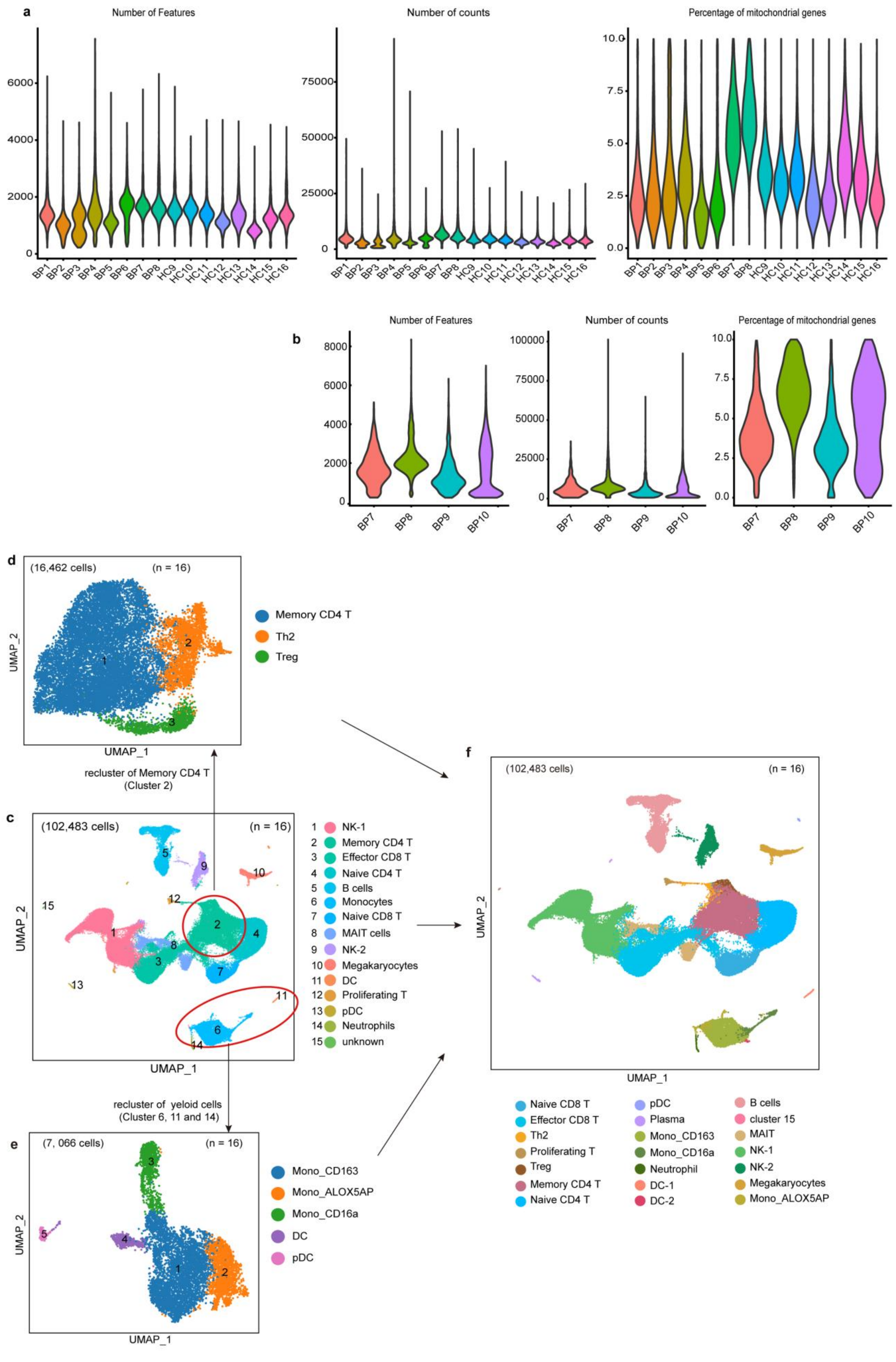

a
Number of Features
Number of counts
Percentage of mitochondrial genes
BP1
BP2
BP3
BP4
BP5
BP6
BP7
BP8
HC9
HC10
HC11
HC12
HC13
HC14
HC15
HC16
b
Number of Features
Number of counts
Percentage of mitochondrial genes
BP7
BP8
BP9
BP10
d
(16,462 cells)
(n = 16)
Memory CD4 T
Th2
Treg
UMAP_1
UMAP_2
recluster of Memory CD4 T
(Cluster 2)
c
(102,483 cells)
(n = 16)
1 NK-1
2 Memory CD4 T
3 Effector CD8 T
4 Naive CD4 T
5 B cells
6 Monocytes
7 Naive CD8 T
8 MAIT cells
9 NK-2
10 Megakaryocytes
11 DC
12 Proliferating T
13 pDC
14 Neutrophils
15 unknown
UMAP_1
UMAP_2
recluster of yeloid cells
(Cluster 6, 11 and 14)
e
(7, 066 cells)
(n = 16)
Mono_CD163
Mono_ALOX5AP
Mono_CD16a
DC
pDC
UMAP_1
UMAP_2
f
(102,483 cells)
(n = 16)
UMAP_1
UMAP_2
Naive CD8 T
Effector CD8 T
Th2
Proliferating T
Treg
Memory CD4 T
Naive CD4 T
pDC
Plasma
Mono_CD163
Mono_CD16a
Neutrophil
DC-1
DC-2
B cells
cluster 15
MAIT
NK-1
NK-2
Megakaryocytes
Mono_ALOX5AP

Supplementary Fig. 10 The quality of PBMC and blister samples, as well as the identification of clusters within PBMC samples.

a Violin plots illustrating the quality control metrics used in the scRNA-seq analysis for each PBMC sample's features, counts, and mitochondrial percentage per cell. **b** Violin plots illustrating the quality control metrics used in the scRNA-seq analysis for each blister sample's features, counts, and mitochondrial percentage per cell. **c** 15 clusters were revealed by PBMCs scRNA-seq data. **d** Sub-clustering of memory CD4 T cells (cluster 2). **e** Sub-clustering of myeloid cells (cluster 6, 11 and 14). **f** 21 clusters were revealed after recluster of memory CD4 T cells and myeloid cells.

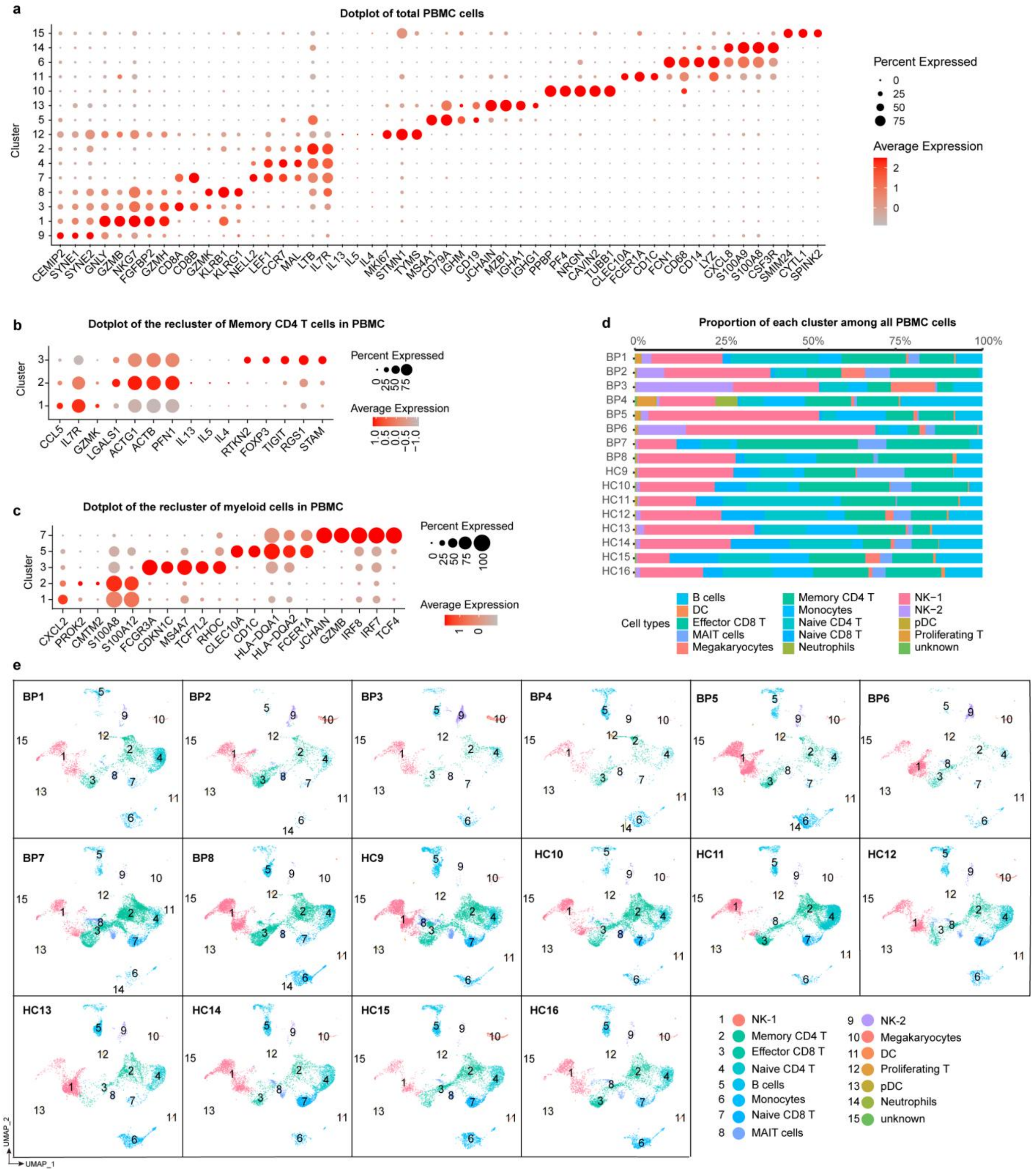

Supplementary Fig. 11 The Dot plot of the signature genes of PBMC clusters and the distribution of clusters within the PBMC samples.

a Dot plot of the signature genes of major cell types depicted in (Fig. S10c). **b** Dot plot of the signature genes of major cell types in CD4 T cells depicted in (Fig. S10d). **c** Dot plot of the signature genes of major cell types in myeloid cells depicted in (Fig. S10e). **d** Bar plots illustrate

the relative contributions of PBMC cell types in each sample. **e** UMAP plot for PBMC cells split by each sample.

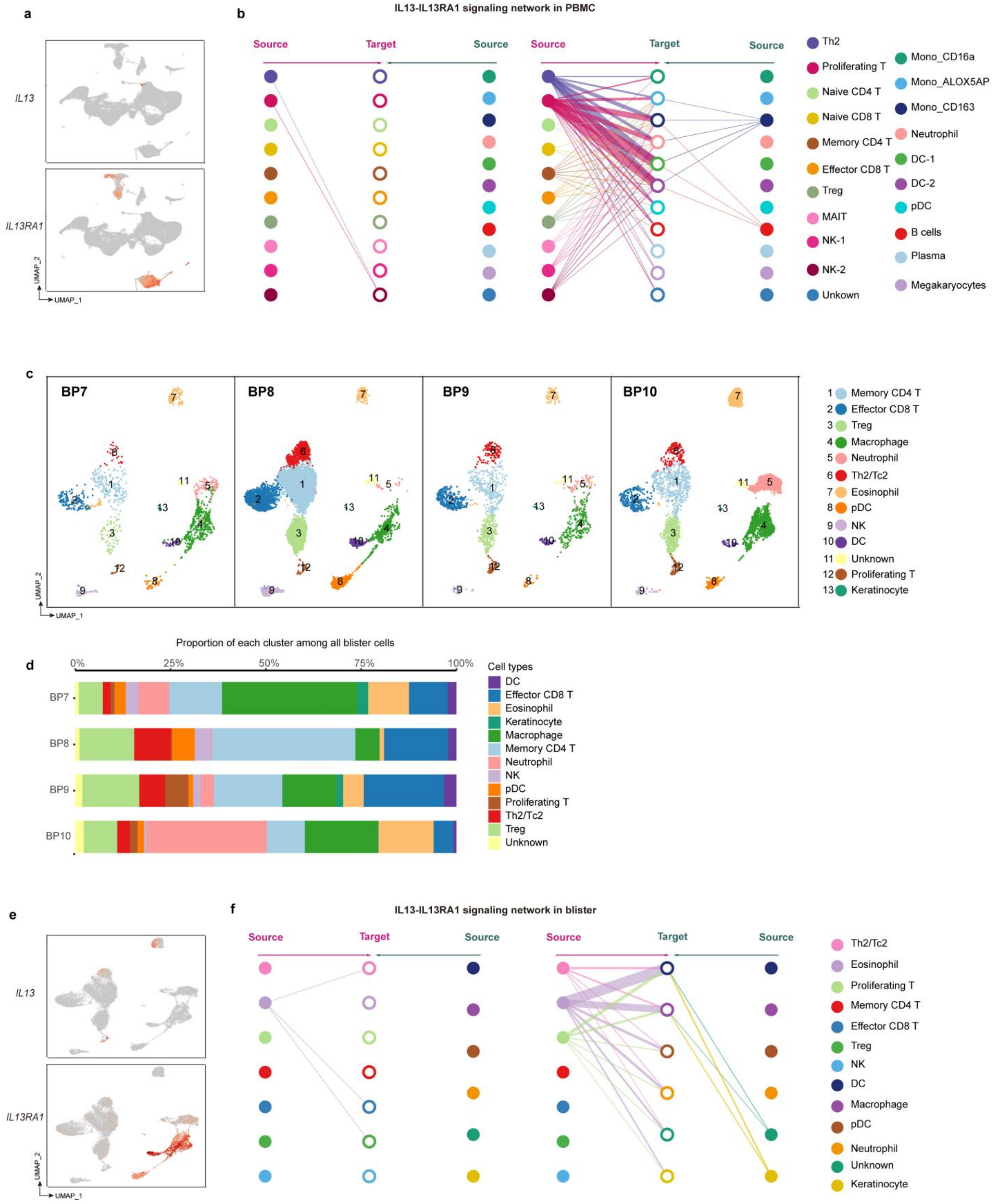

Supplementary Fig. 12 The distribution of clusters in blister samples and the *IL13*-*IL13RA1* ligand-receptor pair in PBMCs and blister.

a UMAP plot of the expression of *IL13* and *IL13RA1* in PBMCs. **b** Hierarchical plot showing inferred intercellular communication network of *IL13-IL13RA1* signaling in PBMCs. **c** UMAP plot for blister cells split by each sample. **d** Bar plots illustrate the relative contributions of blister cell types in each patient. **e** The expression profile of *IL13* and *IL13RA1* in blister cells. **f** Hierarchical plot showing inferred intercellular communication network of *IL13-IL13RA1* signaling in blister cells.

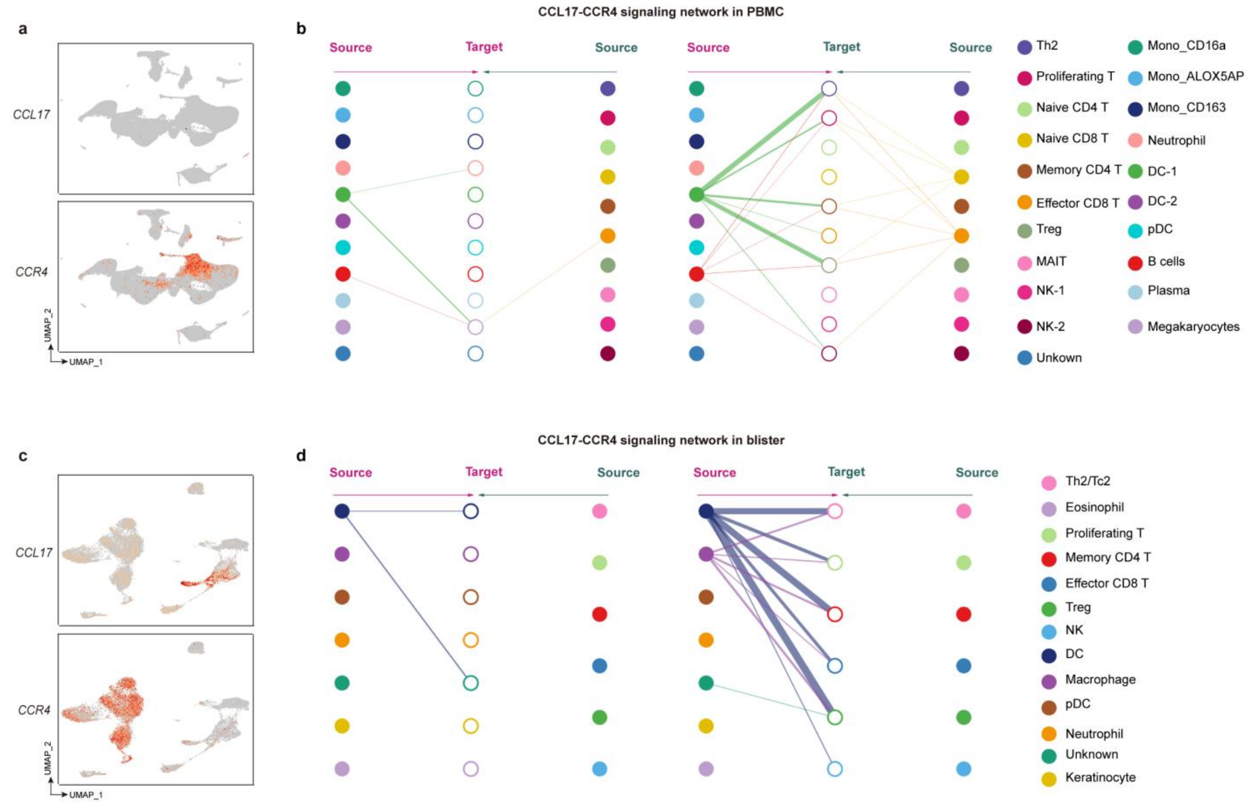

Supplementary Fig. 13 *CCL17*-*CCR4* ligand-receptor pair in PBMC and blister.

a UMAP plot of the expression of *CCL17* and *CCR4* in PBMCs. **b** Hierarchical plot showing inferred intercellular communication network of *CCL17*-*CCR4* signaling in PBMCs. **c** The expression profile of *CCL17* and *CCR4* in blister cells. **d** Hierarchical plot showing inferred intercellular communication network of *CCL17*-*CCR4* signaling in blister cells.

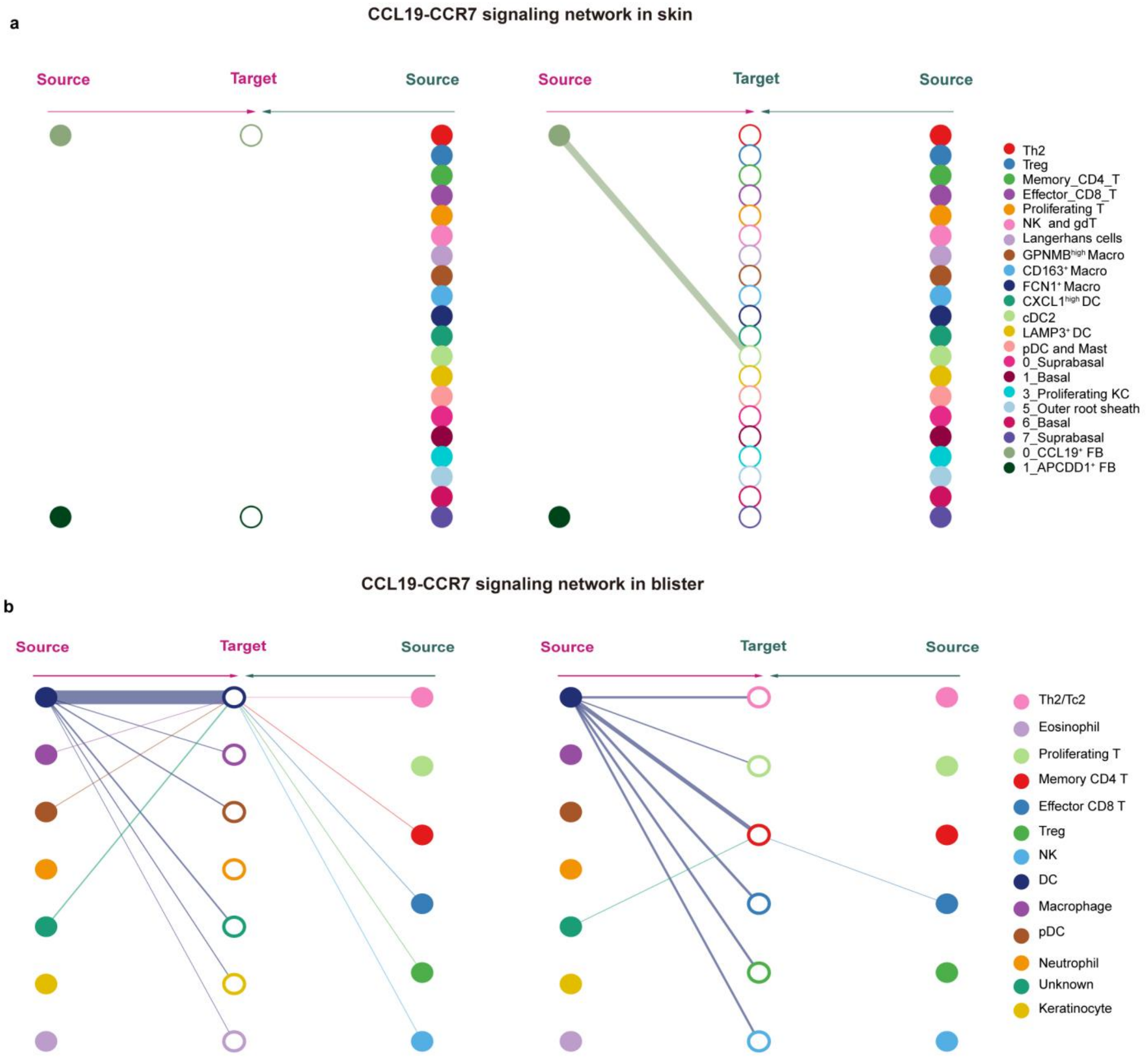

Supplementary Fig. 14 ***CCL19-CCR7*** **ligand-receptor pair in skin and blister samples.**

a-**b** Hierarchical plot showing inferred intercellular communication network of *CCL19-CCR7* signaling in skin (**a**) and blister (**b**) samples.

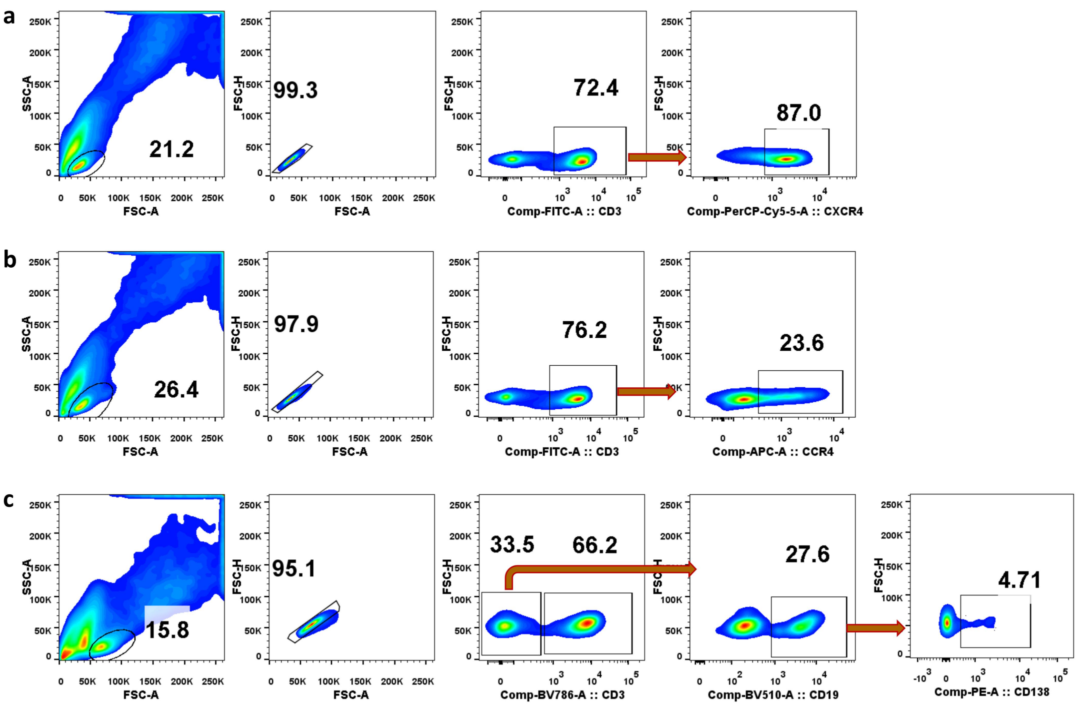

Supplementary Fig. 15 The gating strategy of T cells and B cells to test the expression of CXCR4 (a), CCR4 (b) and CD138 (c).

Supplementary Table 1 The sample information included in the study.

No.	Sex	Age	BP180	BP230	BP180/ BP230	Discovery cohort			Validation cohort 1 (IHC, IF)	Validation cohort 2 (ELISA)	Validation cohort 3 (Flow)
						skin	PBMC	blister			
BP1	Male	81	>200	>200	1	√	√				
BP2	Male	74	>200	133.6	1.5	√	√				
BP3	Female	79	168.5	2.1	80.24	√	√				
BP4	Male	70	>200	0		√	√				
BP5	Male	73	>200	155	1.29	√	√				
BP6	Male	79	85.4	94.9	0.9		√				
BP7	Female	56	>200	0			√	√			
BP8	Female	40	>200	0			√	√			
BP9	Male	72	>200	0				√			
BP10	Male	88	NA	NA				√			
BP11	Male	53	57.2	0					√		√
BP12	Male	68	>200	2.86	69.96				√		√
BP13	Male	50	>200	15.73	12.71				√	√	√
BP14	Male	54	189.5	0					√		√
BP15	Female	91	131.8	155	0.85				√		
BP16	Male	82	>200	94.7	2.11				√		
BP17	Male	82	>200	0					√		
BP18	Male	73	>200	188.8	1.06				√		
BP19	Female	57	>200	0					√	√	√
BP20	Female	53	>200	173.5	1.15				√	√	√
BP21	Female	66	153	0					√		√
BP22	Female	70	>200	0					√	√	√
BP23	Female	70	>200	0						√	
BP24	Male	66	>200	>200	1					√	

BP25	Male	85	>200	>200	1					√	
BP26	Male	73	>200	0						√	
BP27	Male	69	>200	0						√	√
BP28	Male	41	30.79	47.54	0.65					√	
BP29	Male	63	>200	142.21	1.41					√	
BP30	Male	60	145.29	132.73	1.1					√	
BP31	Female	76	166.25	110.51	1.5					√	
BP32	Male	64	>200	5.89	33.96					√	
BP33	Male	77	>200	4.24	47.17					√	
BP34	Female	72	>200	15.89	12.59					√	
BP35	Male	58	25.7	138.12	0.19					√	
BP36	Male	76	87.89	16.07	5.47					√	
BP37	Female	79	2.48	56.43	0.04					√	
BP38	Male	77	105.19	5.46	19.27					√	
BP39	Male	62	1.7	46.16	0.02					√	
BP40	Male	65	11.48	122.87	0.09					√	
BP41	Male	68	>200	0						√	
BP42	Male	78	>200	4.13	48.42					√	
BP43	Female	45	>200	0							√
BP44	Male	72	>200	0							√
BP45	Male	23	>200	0							√
BP46	Male	79	>200	80.9	2.47						√
BP47	Male	36	>200	0							√
BP48	Female	80	>200	0							√
BP49	Male	71	>200	0							√
BP50	Male	79	>200	125.4	1.59						√
BP51	Male	74	>200	50.2	3.98						√
BP52	Male	57	>200	0							√
BP53	Female	51	>200	0							√
BP54	Female	80	>200	>200	1						√

BP55	Male	71	>200	0							√
BP56	Female	71	157.3	0							√
BP57	Male	64	>200	0							√
BP58	Female	77	>200	>200	1				√		
BP59	Female	78	>200	>200	1				√		
BP60	Male	80	>200	>200	1				√		
BP61	Male	53	>200	>200	1				√		
BP62	Male	72	0	148.3	0				√		
BP63	Male	74	6.4	20.1	0.32				√		
BP64	Male	75	19.83	38.97	0.53				√		√
BP65	Male	76	4.86	42.86	0.11				√		√
BP66	Male	81	0	42.3	0				√		
BP67	Female	83	8.8	141.9	0.06				√	√	
BP68	Female	52	0.42	126.52	0.003				√	√	√
BP69	Female	71	6.21	98.24	0.06				√	√	√
BP70	Female	45	0	36.89	0				√	√	
BP71	Female	80	>200	>200	1				√		
BP72	Male	87	179.43	183.9	0.98				√		
BP73	Male	80	>200	>200	1				√		
BP74	Female	75	>200	0					√		
BP75	Female	72	>200	0					√		
BP76	Female	16	0	55.33	0				√		
BP77	Male	78	0	36.99	0					√	
BP78	Male	73	16.76	162.15	0.1					√	
BP79	Female	47	6.9	36.25	0.19					√	√
BP80	Male	49	0	73.65	0					√	
BP81	Male	70	2.16	133.01	0.016					√	
BP82	Female	41	0	74.98	0					√	

BP83	Male	69	2.52	86.4	0.03					√	
BP84	Male	84	0.58	143.81	0.004					√	
BP85	Male	66	1.91	27.65	0.07					√	
BP86	Male	77	>200	4.48	44.64					√	
BP87	Female	65	117.45	4.97	23.63					√	
BP88	Male	40	137.69	0.68	202.49					√	√
BP89	Male	45	137.82	3.16	43.61					√	
BP90	Female	77	88.58	1.01	87.7					√	√
BP91	Female	82	130.32	8.08	16.13					√	
BP92	Female	76	>200	10.92	18.32					√	
BP93	Female	66	106.73	0.63	169.41					√	
BP94	Female	82	199.86	0.93	214.9					√	√
BP95	Female	81	66.91	1.69	39.59					√	
BP96	Male	77	>200	0.91	219.78					√	
BP97	Female	41	55.81	0						√	
BP98	Male	80	>200	11.66	17.15					√	
BP99	Male	80	188.85	1.63	115.86					√	
BP100	Female	86	>200	17.86	11.2					√	√
BP101	Female	66	30.55	3.1	9.85					√	
BP102	Female	74	73.91	1.78	41.52					√	
BP103	Female	71	98.45	1.34	73.47					√	
BP104	Female	72	97.31	0						√	
BP105	Female	66	26.25	3.91	6.71					√	
BP106	Female	66	>200	0						√	
BP107	Male	66	62.34	0.11	566.73					√	
BP108	Female	61	>200	>200	1					√	√
BP109	Male	81	97.78	54.77	1.79					√	
BP110	Male	81	122.93	36.54	3.36					√	

BP111	Female	81	>200	>200	1					√	
BP112	Female	91	>200	>200	1					√	
BP113	Male	75	66.36	48.18	1.38					√	√
BP114	Female	69	>200	68.42	2.92					√	
BP115	Female	80	>200	>200	1					√	
BP116	Female	78	>200	135.48	1.48					√	
BP117	Male	87	179.43	183.9	0.98					√	
BP118	Female	64	13.4	30.13	0.44					√	√
BP119	Male	68	6.21	32.28	0.19						√
BP120	Male	76	8.74	163.33	0.05						√
BP121	Male	77	2.69	21.04	0.13						√
BP122	Male	64	0.21	24.86	0.008						√
BP123	Male	85	>200	193	1.03						√
BP124	Female	80	>200	>200	1						√
BP125	Female	74	49	>200	0.245						√
BP126	Female	75	>200	0							√
BP127	Male	67	>200	4.9	40.8						√
BP128	Female	84	>200	1.67	119.76						√
BP129	Male	68	30.55	2.42	12.62						√
BP130	Female	85	>200	124.77	1.6						√
BP131	Male	46	>200	0							√
BP132	Female	72	>200	0							√
BP133	Female	16	0	55.33	0						√
BP134	Male	74	31.86	0.56	56.89						√
BP135	Male	79	>200	80.9	2.47						√
HC1	Male	17				√					
HC2	Male	51				√					
HC3	Female	52				√					

HC4	Male	52				√					
HC5	Male	27				√					
HC6	Male	34				√					
HC7	Female	54				√					
HC8	Male	16				√					
HC9	Female	40					√				
HC10	Male	45					√				
HC11	Male	53					√				
HC12	Female	27					√				
HC13	Female	55					√				
HC14	Male	26					√				
HC15	Female	39					√				
HC16	Male	38					√				
HC17	Male	58							√		
HC18	Male	45							√		
HC19	Female	70							√		
HC20	Female	51							√		
HC21	Male	46							√		
HC22	Female	73							√		
HC23	Male	58							√		
HC24	Female	60							√		
HC25	Female	71							√		
HC26	Female	73							√		
HC27	Female	58								√	
HC28	Female	59								√	
HC29	Female	55								√	
HC30	Male	54								√	
HC31	Female	59								√	
HC32	Male	58								√	
HC33	Male	53								√	

HC34	Male	50								√	
HC35	Male	58								√	
HC36	Female	37								√	
HC37	Female	45								√	
HC38	Male	34								√	
HC39	Male	39								√	
HC40	Male	47								√	
HC41	Female	35								√	
HC42	Male	32								√	
HC43	Female	28								√	
HC44	Male	30								√	
HC45	Male	43									√
HC46	Male	60									√
HC47	Male	37									√
HC48	Female	35									√
HC49	Female	33									√
HC50	Male	60									√
HC51	Female	71									√
HC52	Male	70									√
HC53	Female	60									√
HC54	Female	30									√
HC55	Male	69									√
HC56	Female	61									√
HC57	Male	37							√		
HC58	Female	55							√		
HC59	Male	57							√		
HC60	Female	61							√		
HC61	Male	59							√		
HC62	Female	40							√		
HC63	Male	32							√		

HC64	Female	73							√		
HC65	Female	76							√		
HC66	Male	58							√		
HC67	Male	33							√		
HC68	Male	58							√		
HC69	Female	57								√	√
HC70	Male	61								√	
HC71	Male	48								√	√
HC72	Female	54								√	
HC73	Male	60								√	
HC74	Female	58								√	√
HC75	Female	51								√	
HC76	Male	57								√	
HC77	Female	60								√	
HC78	Male	71								√	√
HC79	Male	75								√	
HC80	Male	50								√	
HC81	Female	62								√	
HC82	Male	60								√	
HC83	Female	64								√	
HC84	Female	73									√
HC85	Male	72									√
HC86	Male	64									√
HC87	Male	56									√
HC88	Male	60									√
HC89	Female	55									√
HC90	Male	52									√
HC91	Female	48									√
HC92	Female	60									√
HC93	Female	59									√

HC94	Female	48									√
HC95	Male	49									√
HC96	Male	49									√

Supplementary Table 2 The detailed information of antibodies used in the study.

IHC/IF				
Name	Company	Catolog	Clone	Dilution
CD3	CST	85061S	D7A6E	1/200
GATA3	CST	5852S	D13C9	1/200
PDGFRα	CST	3174S	D1E1E	1/200
CD68	CST	76437S	D4B9C	1/200
IL13	Bioss	bs-0560R	polyclonal	1/200
CXCL12/SDF1	CST	97958S	D8G6H	1/200
CXCR4	CST	97680S	D4Z7W	1/200
CD11C	CST	45581S	D3V1E	1/200
PLA2G2A	abcam	AB23705	polyclonal	1/200
CD207	CST	13650S	D9H7R	1/200
**Flow cytometry**				
Name	Company	Catolog	Clone	Dilution
CD3	Biolegend	317330	OKT3	5ul/test
CD19	Biolegend	363024	SJ25C1	5ul/test
CD138	Biolegend	352306	DL-101	5ul/test
CXCR4	Biolegend	306516	12G5	5ul/test
CCR4	Biolegend	359408	L291H4	5ul/test
