# Supplementary material for: Single-cell transcriptomics analysis of bullous pemphigoid unveils immune-stromal crosstalk in type 2 inflammatory disease
Source: Nat Commun. 2024 Jul 15;15:5949. doi: 10.1038/s41467-024-50283-3 (PMC11251189; doi:10.1038/s41467-024-50283-3)
Supplement: Supplementary file 3 — Description of Additional Supplementary Files [file 41467_2024_50283_MOESM3_ESM.pdf]

Description of Additional Supplementary Files

Supplementary Data 1. The Excel file includes Metadata of all cells from 13 scRNA-seq skin samples.

Supplementary Data 2. The Excel file contains marker genes of clusters derived from all skin cells.

Supplementary Data 3. The Excel file comprises Metadata pertaining to the subclusters of all immune cells derived from 13 scRNA-seq skin samples.

Supplementary Data 4. The Excel file includes marker genes of clusters derived from all immune cells across 13 scRNA-seq skin samples.

Supplementary Data 5. The Excel file comprises Metadata pertaining to the subclusters of all fibroblasts cells derived from 13 scRNA-seq skin samples.

Supplementary Data 6. The Excel file includes marker genes of clusters derived from all fibroblasts cells across 13 scRNA-seq skin samples.

Supplementary Data 7. The Excel file comprises Metadata pertaining to the subclusters of all keratinocytes cells derived from 13 scRNA-seq skin samples.

Supplementary Data 8. The Excel file includes marker genes of clusters derived from all keratinocytes cells across 13 scRNA-seq skin samples.

Supplementary Data 9. The Excel file includes differentially expressed genes between BP and controls in *CCL19*+ FB cluster.

Supplementary Data 10. The Excel file includes differentially expressed genes between BP and controls in *APCDD1*+ FB cluster.

Supplementary Data 11. The Excel file includes Metadata of all cells from 16 scRNA-seq PBMC samples.

Supplementary Data 12. The Excel file contains marker genes of clusters derived from all PBMC cells.

Supplementary Data 13. The Excel file includes Metadata of all cells from 4 scRNA-seq blister samples.

Supplementary Data 14. The Excel file contains marker genes of clusters derived from all blister cells.

Supplementary Data 15. The Excel file contains information of pathways in figure 3a from CellChat database.
