# Supplementary material for: Single-cell transcriptomics analysis of bullous pemphigoid unveils immune-stromal crosstalk in type 2 inflammatory disease
Source: Nat Commun. 2024 Jul 15;15:5949. doi: 10.1038/s41467-024-50283-3 (PMC11251189; doi:10.1038/s41467-024-50283-3)
Supplement: Supplementary file 4 — Supplementary Data 1-15 [file 41467_2024_50283_MOESM4_ESM.zip › Supplementary Data/Description of Additional Supplementary Data files.docx]

**Description of Additional Supplementary Data Files**

**Supplementary Data 1**. The Excel file includes Metadata of all cells from 13 scRNA-seq skin samples.

**Supplementary Data 2**. The Excel file contains marker genes of clusters derived from all skin cells.

**Supplementary Data 3**. The Excel file comprises Metadata pertaining to the subclusters of all immune cells derived from 13 scRNA-seq skin samples.

**Supplementary Data 4**. The Excel file includes marker genes of clusters derived from all immune cells across 13 scRNA-seq skin samples.

**Supplementary Data 5**. The Excel file comprises Metadata pertaining to the subclusters of all fibroblasts cells derived from 13 scRNA-seq skin samples.

**Supplementary Data 6**. The Excel file includes marker genes of clusters derived from all fibroblasts cells across 13 scRNA-seq skin samples.

**Supplementary Data 7**. The Excel file comprises Metadata pertaining to the subclusters of all keratinocytes cells derived from 13 scRNA-seq skin samples.

**Supplementary Data 8**. The Excel file includes marker genes of clusters derived from all keratinocytes cells across 13 scRNA-seq skin samples.

**Supplementary Data 9**. The Excel file includes differentially expressed genes between BP and controls in *CCL19*+ FB cluster.

**Supplementary Data 10**. The Excel file includes differentially expressed genes between BP and controls in *APCDD1*+ FB cluster.

**Supplementary Data 11**. The Excel file includes Metadata of all cells from 16 scRNA-seq PBMC samples.

**Supplementary Data 12**. The Excel file contains marker genes of clusters derived from all PBMC cells.

**Supplementary Data 13**. The Excel file includes Metadata of all cells from 4 scRNA-seq blister samples.

**Supplementary Data 14**. The Excel file contains marker genes of clusters derived from all blister cells.

**Supplementary Data 15**. The Excel file contains information of pathways in figure 3a from CellChat database.
