# Supplementary material for: Single-cell transcriptomics analysis of bullous pemphigoid unveils immune-stromal crosstalk in type 2 inflammatory disease
Source: Nat Commun. 2024 Jul 15;15:5949. doi: 10.1038/s41467-024-50283-3 (PMC11251189; doi:10.1038/s41467-024-50283-3)
Supplement: Supplementary file 5 — Reporting Summary [file 41467_2024_50283_MOESM5_ESM.pdf]

nature portfolio

Corresponding author(s): Furen Zhang, Hong Liu

Last updated by author(s): Jun 12, 2024

Reporting Summary

Nature Portfolio wishes to improve the reproducibility of the work that we publish. This form provides structure for consistency and transparency in reporting. For further information on Nature Portfolio policies, see our Editorial Policies and the Editorial Policy Checklist.

Statistics

For all statistical analyses, confirm that the following items are present in the figure legend, table legend, main text, or Methods section.

n/a	Confirmed	
☐	☒	The exact sample size (*n*) for each experimental group/condition, given as a discrete number and unit of measurement
☐	☒	A statement on whether measurements were taken from distinct samples or whether the same sample was measured repeatedly
☐	☒	The statistical test(s) used AND whether they are one- or two-sided<br>*Only common tests should be described solely by name; describe more complex techniques in the Methods section.*
☐	☒	A description of all covariates tested
☐	☒	A description of any assumptions or corrections, such as tests of normality and adjustment for multiple comparisons
☐	☒	A full description of the statistical parameters including central tendency (e.g. means) or other basic estimates (e.g. regression coefficient) AND variation (e.g. standard deviation) or associated estimates of uncertainty (e.g. confidence intervals)
☐	☒	For null hypothesis testing, the test statistic (e.g. *F*, *t*, *r*) with confidence intervals, effect sizes, degrees of freedom and *P* value noted<br>*Give P values as exact values whenever suitable.*
☒	☐	For Bayesian analysis, information on the choice of priors and Markov chain Monte Carlo settings
☒	☐	For hierarchical and complex designs, identification of the appropriate level for tests and full reporting of outcomes
☐	☒	Estimates of effect sizes (e.g. Cohen's *d*, Pearson's *r*), indicating how they were calculated

Our web collection on statistics for biologists contains articles on many of the points above.

Software and code

Policy information about availability of computer code

Data collection	No software was used
Data analysis	All softwares are commercially or freely available.<br>Software, tools, algorithms and packages (including number versions) used in the study are listed hereafter:<br>CellRanger software suite (v 6.1.2); human reference genome GRCh38-2020-A; Seurat R package (v 4.0.0); rCASC (v 5.5.6); Scrublet R package (v0.2.3); CellChat R package (v 1.5.0)

For manuscripts utilizing custom algorithms or software that are central to the research but not yet described in published literature, software must be made available to editors and reviewers. We strongly encourage code deposition in a community repository (e.g. GitHub). See the Nature Portfolio guidelines for submitting code & software for further information.

Data

Policy information about availability of data

All manuscripts must include a data availability statement. This statement should provide the following information, where applicable:

- Accession codes, unique identifiers, or web links for publicly available datasets
- A description of any restrictions on data availability
- For clinical datasets or third party data, please ensure that the statement adheres to our policy

The raw scRNA-seq data of 33 samples in fastq format generated in this study have been deposited in the Genome Sequence Archive (GSA) database under

accession code HRA005922, HRA003993, HRA000145, HRA000471, HRA000847, HRA005913 and HRA003995 [https://ngdc.cncb.ac.cn/gsa-human]. Among these, twenty-five samples are new additions, including four blisters and eight PBMCs from BP patients (HRA005922), five lesions from BP patients (HRA003993), three samples of normal skin from HC (HRA003995), and five PBMCs from HC (HRA005913). Furthermore, eight samples have been previously reported: three samples of normal skin from HC (HRA000145), two more samples of normal skin from HC (HRA000471), and three PBMCs from HC (HRA000847). The scRNA-seq data of these 33 samples in 10X Genomics format have been also deposited in Zenodo database under accession code 10924853 [https://zenodo.org/records/10924853]. These data are freely available without any restrictions or time limits. Any questions or additional requests can be directed to the corresponding authors. Source data are provided with this paper.

Representative code is available on GitHub (https://github.com/zzwang1030/scRNA_BP, DOI: 10.5281/zenodo.11567155). This code is openly available with no restriction or time limit. Any queries or further requests can be addressed to the corresponding authors.

Research involving human participants, their data, or biological material

Policy information about studies with human participants or human data. See also policy information about sex, gender (identity/presentation), and sexual orientation and race, ethnicity and racism.

Reporting on sex and gender	Self-reported sex of participants has been reported in the manuscript. We provided detailed messages of gender in supplementary table S1.
Reporting on race, ethnicity, or other socially relevant groupings	All individuals were of Chinese descent, which has been specified in the manuscript.<br>We collected the age, sex and other clinical information of all samples in Table S1.
Population characteristics	This study included 135 patients and 96 controls, with ages ranging from 16 to 91 years old. All the information including age, sex and other clinical information of all samples are shown in Table S1.
Recruitment	The patients and controls were enrolled from Hospital for Skin Diseases, Shandong First Medical University. Diagnosis of BP relied on typical clinical and histological presentations, alongside direct or indirect immunofluorescence examinations.
Ethics oversight	All procedures involving human tissue samples were approved by Shandong Provincial Institute of Dermatology and Venereology.

Note that full information on the approval of the study protocol must also be provided in the manuscript.

Field-specific reporting

Please select the one below that is the best fit for your research. If you are not sure, read the appropriate sections before making your selection.

- [x] Life sciences
- [ ] Behavioural & social sciences
- [ ] Ecological, evolutionary & environmental sciences

For a reference copy of the document with all sections, see nature.com/documents/nr-reporting-summary-flat.pdf

Life sciences study design

All studies must disclose on these points even when the disclosure is negative.

Sample size	All samples were divided into two groups: the control group (n=135) and the BP group (n= 96). We provided detailed messages of sample size in Figure 1a.
Data exclusions	All samples obtained from patients and healthy controls enrolled in the study were included in the analysis. No outliers were excluded.
Replication	We have biological replicates for all the procedures shown through the manuscript.<br>All samples within the same group were considered biological replicates.
Randomization	Not applicable, as no comparison of experimental groups is performed. All comparisons presented are performed on cell groups from different disease conditions or different cell types.
Blinding	Blinding is not be applicable in the context of scRNA-seq due to the technical complexity and exploratory nature of the techniques.

Reporting for specific materials, systems and methods

We require information from authors about some types of materials, experimental systems and methods used in many studies. Here, indicate whether each material, system or method listed is relevant to your study. If you are not sure if a list item applies to your research, read the appropriate section before selecting a response.

Materials & experimental systems

n/a	Involved in the study
[ ]	[x] Antibodies
[ ]	[x] Eukaryotic cell lines
[x]	[ ] Palaeontology and archaeology
[x]	[ ] Animals and other organisms
[x]	[ ] Clinical data
[x]	[ ] Dual use research of concern
[x]	[ ] Plants

Methods

n/a	Involved in the study
[x]	[ ] ChIP-seq
[ ]	[x] Flow cytometry
[x]	[ ] MRI-based neuroimaging

Antibodies

Antibodies used

The following antibodies for IHC or IF were diluted as followed:
CD3,CST,85061S,CloneD7A6E,1:200
GATA3,CST,5852S,CloneD13C9,1:200
PDGFR,CST,3174S,CloneD1E1E,1:200
CD19,abcam,AB134114,CloneEPR5906,1:200
CD68,CST,76437S,CloneD4B9C,1:200
IL13,Bioss,bs-0560R, polyclonal, 1:200
CXCL12/SDF1,CST,97958S,CloneD8G6H,1:200
CXCR4,CST,97680S,CloneD4Z7W,1:200
CD138,AB128936,CloneEPR6454,1:200
CD11C,CST,45581S,CloneD3V1E,1:200
PLA2G2A,abcam,AB23705,polyclonal,1:200
MPO,CST,14569S,E1E7I,1:200
Tryptase,CST,19523S,CloneE7M2U,1:200

The following antibodies for flow cytometry were diluted as followed:
CD3,Biolegend,317330,CloneOKT3, 5ul/test
CD19,Biolegend,363024,CloneSJ25C1, 5ul/test
CXCR4,Biolegend,306516,Clone12G5, 5ul/test
CCR4,Biolegend,359408,CloneL291H4, 5ul/test
CD138,Biolegend,352306,CloneDL-101, 5ul/test

Validation

Validation statements on the manufacturers' website.

Eukaryotic cell lines

Policy information about cell lines and Sex and Gender in Research

Cell line source(s)

THP-1 and Jurkat cells were obtained from ATCC

Authentication

THP-1 and Jurkat cells were authenticated

Mycoplasma contamination

All the cells used in this study were tested Mycoplasma-negative.

Commonly misidentified lines
(See ICLAC register)

No commonly misidentified cell lines were used in this study.

Plants

Seed stocks

Not applicable

Novel plant genotypes

Not applicable

Authentication

Not applicable

Flow Cytometry

Plots

Confirm that:

- [x] The axis labels state the marker and fluorochrome used (e.g. CD4-FITC).
- [x] The axis scales are clearly visible. Include numbers along axes only for bottom left plot of group (a 'group' is an analysis of identical markers).
- [x] All plots are contour plots with outliers or pseudocolor plots.
- [x] A numerical value for number of cells or percentage (with statistics) is provided.

Methodology

Sample preparation	The PBMCs were incubated with surface antibodies (CD3, CD19, CXCR4, CCR4, and CD138) in a staining buffer for 30 minutes at 4 °C.
Instrument	FACSAria Fusion flow cytometer (BD Biosciences)
Software	FlowJo 10.8.1 Software
Cell population abundance	T cells and B cells from PBMC were analyzed.
Gating strategy	Cells were selected according to the FCS-A and SSC-A to exclude debris.<br>Duplets were removed with the FCS-A and FSC-H gating.<br>T cells were gated as as CD3+ cells<br>B cells were gated as CD3-CD19+ cells.

- [x] Tick this box to confirm that a figure exemplifying the gating strategy is provided in the Supplementary Information.
